# Oriental schistosomiasis with neurological complications: case report

**DOI:** 10.1186/1476-0711-10-5

**Published:** 2011-02-07

**Authors:** Yuesheng Li, Allen G Ross, Xunya Hou, Zhaoyang Lou, Donald P McManus

**Affiliations:** 1 Hunan Institute of Parasitic Diseases, World Health Organisation Collaborating Centre for Research and Control on Schistosomiasis in Lake Region, Yueyang, Hunan, People's Republic of China; 2 Molecular Parasitology Laboratory, Infectious Diseases Division, Queensland Institute of Medical Research, Herston, Brisbane, Queensland, Australia; 3 School of Public Health, Griffith University, Meadowbrook, Australia; 4 Griffith Institute of Health, Griffith University, Australia

## Abstract

We describe a case of cerebral schistosomiasis, caused by Asian (oriental) *Schistosoma japonicum *trematode blood flukes, in a young Chinese patient and its management. We also provide a brief update for physicians on the clinical features, diagnosis and treatment of schistosomiasis, with particular emphasis on neuroschistosomiasis, the most severe clinical outcome associated with this parasitic infection.

## Background

Although not commonly reported in western countries, schistosomiasis is, after malaria, the second most prevalent tropical disease, affecting over 200 million people [1]. It is an intravascular disease, caused by dioecious parasitic trematode worms of the genus *Schistosoma*. Eggs released by adult female worms cause the majority of lesions in schistosomiasis when they embolize in the liver, spleen, lungs, or urino-genital or cerebrospinal systems [1-3]. Egg secretions provoke eosinophilic inflammatory and granulomatous reactions which are progressively replaced by fibrotic deposits [1,2]. Schistosomiasis is thus associated with chronic liver and intestinal or genitourinary tract fibrosis. Neurological complications can also develop, and neuroschistosomiasis is the most severe clinical outcome associated with schistosome infection [1,2]. Neurological complications during the early course of infection are thought to occur through *in situ *egg deposition following aberrant migration of adult worms to the brain or spinal cord [1,2]. The presence of eggs in the central nervous system (CNS) induces a Th2 driven periovular granuloma reaction [1]. The mass effect of thousands of eggs and the large granulomas concentrated within the brain or spinal cord explain the signs and symptoms of increased intracranial pressure, focal neuropathy and subsequent clinical sequel associated with neuroschistosomiasis [1].

The Asian schistosome species (*S. japonicum; S. mekongi) *lay up to 3000 eggs daily with the resulting morbidity being more severe than with the African forms (*S. mansoni*; *S. haematobium; S. intercalatum*), which produce ten times fewer eggs per day [1,2]. Myelopathy (acute transverse myelitis and subacute myeloradiculopathy) of the lumbosacral region is the most common neurological manifestation of *S. mansoni *or *S. haematobium *infection, whereas acute encephalophalitis of the cortex, subcortex, basal ganglia or internal capsule is typical of *S. japonicum *[1,2].

Only a minority (<5%) of patients will develop CNS symptoms due to schistosomiasis, with cerebral complications being more prevalent than spinal [1]. Cerebral complications include: headache, visual impairment, delirium, seizures, motor deficits and ataxia, whereas, spinal symptoms comprise: lumbar pain, lower limb radicular pain, muscle weakness, sensory loss and bladder dysfunction [1,2]. The onset of neurological symptoms usually takes place within weeks to months, and progress in an acute or subacute manner with the symptoms and signs of the disease worsening over time [1,2].

We describe the diagnosis of a young Chinese schistosomiasis patient with cerebral involvement and its successful management, and briefly review the clinical features, diagnosis and treatment of the disease.

## Case presentation

**Figure 1:**
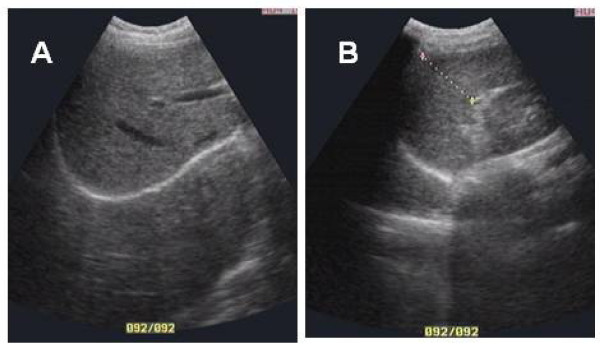
**Ultrasound examination of the patient's liver (A) and spleen (B)**.

**Figure 2:**
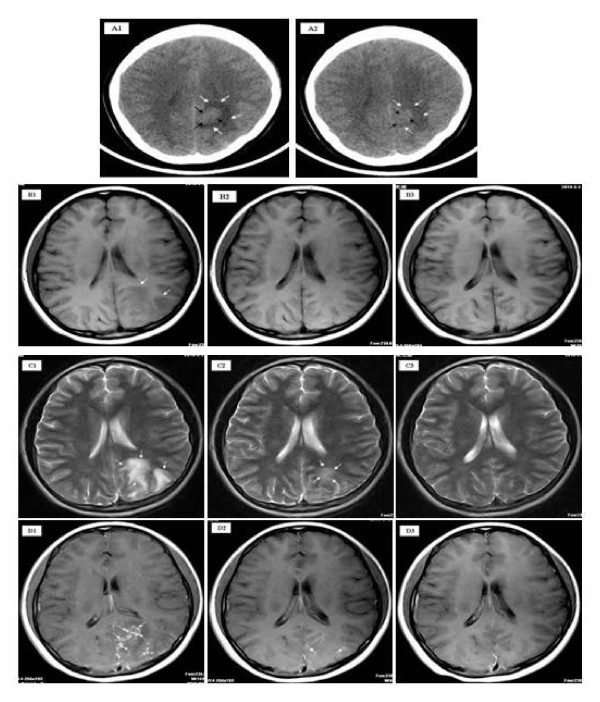
**Unenhanced axial section CT scan of the patient's brain **(A). A 1.6 × 2.4 cm isodensity mass (black arrows) in the left parietal lobe with edema (white arrows) surrounding (A1) prior to praziquantel (PZQ)-treatment. Marked decrease in the size of the mass (black arrows) and reduced edema (white arrows) were evident 21 days post-PZQ treatment (A2). Axial section MRI of the patient's brain (B,C). Hypointensity lesions (white arrows) on T_1_-weighted spin-echo images pre-PZQ treatment (B1) were almost absolved at 25 days post-PZQ treatment (B2) and absorbed at 3 months post-PZQ treatment (B3). Hyperintensity lesions on T_2_-weighted spin-echo images prior to treatment with PZQ (white arrows) (C1) were decreased in size 25 days post-PZQ treatment (white arrows) (C2) and were almost totally resolved at 3 months post-PZQ treatment (C3). Enhanced MRI of the patient's brain (D). Multiple small nodules (white arrows) scattered or clustered at the cortical/subcortical area following intravenous administration of gadolinium-DTPA pre-PZQ treatment (D1) and two nodular enhancements (white arrows) observed at 25 days post-PZQ treatment (D2); almost all nodules were absolved at 3 months post-PZQ treatment (D3).

A 14-year-old female presented to Xiang-Yue hospital, Yueyang City, Hunan Province, People's Republic of China with a four month history of vertigo, headache, vomiting and syncope. The patient had lived in the Dongting Lake region, a highly endemic area for schistosomiasis, since birth. The patient reported no past medical history of epilepsy, tuberculosis, hepatitis B/C and no known drug allergies. Upon physical examination, the patient's vital signs were within normal limits; she had no evidence of skin itching, no fever, no bloody stool, no obvious abdominal discomfort was observed and no other neurological signs were detected. Electrocardiography and chest X-ray were unremarkable. Hematological testing revealed evidence of eosinophilia (eosinophil count 0.8 × 10^9)^ but white blood cells (white blood cell count, 5.6 × 10^9^; lymphocyte count 1.2 × 10^9^; neutrophil count 3.4 × 10^9^), liver and renal functions were within normal limits. Serological tests were positive for *S. japonicum *infection both by indirect haemagglutination and enzyme linked immunosorbent assays, using soluble egg antigen. *S. japonicum *eggs were further detected in the stool by Kato-Katz thick smear stool examination confirming the diagnosis. Ultrasound examination showed a wide echo dot pattern and increased echogenicity but no network in the liver (Figure 1A), and no abnormality in the size or texture of the spleen (Figure 1B). An unenhanced axial section computed tomography (CT) brain scan showed a 1.6 × 2.4 cm isodensity mass in the left parietal lobe with edema (Figure 2A1). Axial section magnetic resonance imaging (MRI) showed two hypointensity and hyperintensity lesions on T_1_- (Figure 2B1) and on T_2_-weighted spin-echo images (Figure 2C1), respectively. Multiple small nodular or 'silt-like' enhancements clustered in the sub-cortical region were evident following intravenous administration of Gadolinium-DTPA (Figure 2D1). A diagnosis of 'cerebral schistosomiasis' was subsequently made and the patient was treated intravenously with 20% mannitol (125 ml daily for 5 days) to lower the intracranial pressure and orally with praziquantel (PZQ) (120 mg/kg three times a day for six days after meal). Follow-up MRI and CT scans (25 days post PZQ treatment) showed that the edema and lesions were partially resolved and were almost completely resolved 3 months post PZQ (Figure 2D3).

## Conclusions

Schistosome infection can provoke an urticarial rash that often persists for days as a maculopapular lesion [4]. Katayama syndrome (acute schistosomiasis) is a systemic hypersensitivity reaction against the migrating parasite or its eggs [1,4]. In many cases, acute infections are asymptomatic. Symptoms that manifest result from an allergic reaction during larval migration and early oviposition by adult worms [4]. Disease onset is usually sudden, producing many non-specific symptoms, such as fever, fatigue, myalgia, malaise, urticaria, non-productive cough, eosinophilia, and patchy pulmonary infiltrates on chest radiograph [1,5]. Abdominal symptoms may develop within a few weeks, because of migration of juvenile worms and egg deposition of the mature worms [5]. High-grade nocturnal fever and eosinophilia are generally present [5]. Most patients recover spontaneously after 2-10 weeks, whereas some develop persistent and more serious disease with weight loss, dyspnoea, diarrhoea, diffuse abdominal pain, hepatomegaly, and generalized rash [4].

Neurological disease resulting from schistosome infection is thought to occur through egg embolism or aberrant migration of adult worms to the brain or spinal cord [6,7]. Central nervous system (CNS) involvement has been described in soldiers and aid workers serving in areas where schistosomiasis is endemic [6-8], and in tourists who have had relatively limited exposure to such areas or the parasite [1]. Focal or generalized tonic-clonic epilepsy is a typical presentation for *S. japonicum *infection with CNS involvement [1]. Focal neurologic deficits may also occur. Among groups of Chinese adults hospitalized with schistosomiasis, up to 4.3% were shown to have CNS disease [9]. The prevalence of epilepsy in communities where infections have occurred has been estimated at 1-4% which is eight times higher than that of the general population [9,10].

Routine screening of patients following freshwater exposure to schistosomiasis should consist of a full blood count, absolute eosinophil count, serology (particularly the presence of IgE antibodies), and faecal microscopy. The detection of schistosome eggs in urine or in faeces by the rapid, simple and inexpensive Kato-Katz thick-smear stool examination is diagnostic [1,2]. Commercially available immunodiagnostic kits are generally not as sensitive as multiple faecal examinations and are less specific, due to cross-reactivity with other helminths. Most techniques detect IgG, IgM or IgE against soluble worm antigen or crude egg antigen by ELISA, indirect hemagglutination or immunofluorescence [1,2].

Praziquantel is the foremost-prescribed drug and is highly effective against the adult worms of all *Schistosoma *species. It is usually augmented by a course of corticosteroids and anticonvulsants in patients presenting with neurological complications [1,3]. A derivative of the antimalarial drug, artemisinin (artemether), has been shown effective against young schistosome parasites[11].

Schistosomiasis is potentially a public health risk to those travelling to endemic areas within Africa and Asia who may be accidentally exposed to infection though contact with cercariae in freshwater canals, lakes, rivers, or springs. Schistosomiasis is not a notifiable disease in many developed countries, including the USA, so there is no accurate information about infection rates among returned travelers and immigrants [1]. Patients can present with neurological complications. When symptomatic, neuroschistosomiasis (NS) is the most severe presentation of schistosome infection; cerebral invasion is mostly caused by *S. japonicum*, with spinal cord involvement due mainly to *S. mansoni *or *S. haematobium *[7]. The chief neurological feature is diffuse encephalopathy and seizures [12,13]. Patients typically present with any of the following symptoms: headache, papilledema, nystagmus, speech disturbances, some degree of motor weakness (hemiplegia, paraplegia or quadriplegia), and cranial nerve abnormalities due to the formation of mass granulomatous lesions and increased intracranial pressure in the cortex, subcortex, basal ganglia or internal capsule [12,13].. Focal or generalized tonic-clonic epilepsy is a typical presentation for *S. japonicum *infection with CNS involvement. Cerebral involvement due to *S. mansoni *or *S. haematobium *typically involves the cerebral and cerebellar cortex and leptomeninges, with myelopathy, such as transverse myelitis, being the most common neurologic manifestation [12,13]. Myleoradiculopathy occurs far less frequently than the cerebral form of the disease [12,13]. Patients with spinal schistosomiasis usually present with lumbar pain, lower limb radicular pain, muscle weakness, sensory loss and bladder dysfunction due to egg deposition and granuloma formation in the spinal cord or cauda equina. This is typically seen in *S. mansoni *and *S. haematobium *infections but is less frequent in *S. japonicum *patients [12,14]. Spinal schistosomiasis can also present as a progressive paraparesis mimicking a spinal cord neoplasm [12]. Cognitive impairment and memory loss deficits have also been described among children infected with *S. japonicum *and *S. mansoni *[15,16].

The true prevalence of neuroschistosomiasis is yet unknown and is presently estimated at between 1-5% of all diagnosed cases of schistosomiasis. There is also a lack of clinical data regarding the timing of treatment, results of surgery and combination therapy [12]. A complete or partial recovery occurs in 70% of patients with myeloradiculopathy [14], but a less favorable outcome is seen for those treated for transverse myelitis. Earlier diagnosis and prompt treatment should improve the prognostic outlook.

## Consent

Written informed consent was obtained from the patient for publication of this case report and any accompanying images. A copy of the written consent is available for review by the Editor-in-Chief of this journal.

This research and the report of the case were approved by the Hunan Institute of Parasitic Diseases Human Research Ethics Committee. As the patient was a minor, written consent was provided by the father as next of kin.

## Competing interests

The authors declare that they have no competing interests.

## Authors' contributions

YSL made substantial contributions to the conception and design of the manuscript, the acquisition of the data and drafted the paper; AGR participated in the design of the study and in the drafting of the paper; XYH treated and followed up the patient; ZYL undertook the CT examination and analyzed the photographs; DPM revised and finalized the manuscript. All authors have read and approved the final manuscript.
